# *C. difficile* associated diarrhoea-don’t blame community or norovirus

**DOI:** 10.1186/1753-6561-5-S6-P186

**Published:** 2011-06-29

**Authors:** N Damani, R Trudy, M Markey, S Wallace

**Affiliations:** 1 Infection Prevention and Control, Craigavon Area Hospital, Portadown, UK

## Introduction / objectives

*C. difficile* associated diarrhoea (CDAD) has been recognised as the most common cause of hospital acquired diarrhoea. At the beginning of 2008, our Trust’s Infection Prevention and Control developed a comprehensive intervention plan to reduce the number of CDAD cases.

## Methods

Implementation of multi-modal intervention which included, early diagnosis, prompt isolation, introduction of hand hygiene, implementation of antibiotic stewardship programme, education and training, enhanced environmental cleaning, and feed back of surveillance (outcome and process) proved very effective in reducing the total no. of CDAD.

## Results

As a result of multi-modal interventions, the total no. of CDAD case were reduced from 286 in 2008 to only 38 cases in 2010. We also carried out Root Cause Analysis of all cases of CDAD and found that only 15 % cases were ‘true’ community cases. In addition, during the winter of 2009, our wards were affected with outbreaks of norovirus and we tested all norovirus outbreak specimens for *C.difficile* toxin. However, unlike previous years, we did not see a rise in the number of new cases of *C.difficile* infection and for the first time, we were not able to find even a single case that was positive both for norovirus and *C.difficile* toxin.

## Conclusion

We concluded that the health care facilities are the major sources of CDAD. We conclude that once you start to control CDAD in hospitals, the number of cases of *C difficile* will reduce both in hospital and community. We also conclude that isolation of *C.difficile* toxin from specimens submitted for norovirus outbreak may detect patient with *C.difficile* colonization ^1^.

1. Damani NN, Wallace S. Does viral gastroenteritis really increase the reports of *Clostridium difficile* infection? *J of Hosp infect* 2011; 77; 171-172.

## Disclosure of interest

None declared.

